# Somatostatin Receptor Scintigraphy Findings in a Patient with Metastatic Gastrinoma and MEN 1 Syndrome

**DOI:** 10.4274/MIRT.021482

**Published:** 2011-12-01

**Authors:** Gözde Mütevelizade, Mehmet Aydın, Ahmet Sezer

**Affiliations:** 1 Baskent University Department of Nuclear Medicine, Ankara, Turkey; 2 Baskent University Department of Oncology, Ankara, Turkey

**Keywords:** Radionuclide imaging, gastrinoma, neoplasm metastasis, multiple endocrine neoplasia Type 1

## Abstract

Liver metastases from neuroendocrine tumours frequently occur and significantly worsen their prognosis. Somatostatin receptor scintigraphy (SRS) is a valuable method for the detection of somatostatin receptor-positive lesions like gastrinoma. In this case report, the importance of SRS to localize the primary tumor and the spread of disease is emphasized in a patient with neuroendocrine liver metastases. A 45-year-old man was admitted to hospital with multiple liver metastasis of neuroendocrine carcinoma. Somatostatin receptor scintigraphy showed multiple intense radiotracer uptakes in the liver and a focal tracer uptake at the right side of the upper abdominal region corresponding to duodenum or pancreas. Elevated serum gastrin levels confirmed the gastrinoma diagnosis.

**Conflict of interest:**None declared.

## INTRODUCTION

Liver metastases from neuroendocrine tumours occur in up to 75% of the patients and significantly worsen their prognosis (1). The imaging techniques to detect primary and metastatic neuroendocrine tumors are computed tomography (CT), magnetic resonance (MR), endoscopic ultrasonography (EUS) and radiolabeled somatostatin analogs.

Multiple endocrine neoplasia type 1 (MEN 1) is a familial syndrome with multiple tumors of parathyroid glands, neuroendocrine system and anterior pituitary gland. The most common endocrine tumors are parathyroid tumors (2). The most frequently seen well- differentiated endocrine tumor of the gastro-entero- pancreatic tract is gastrinoma. Most gastrinomas are located at the right of the superior mesenteric vessels within the head of the pancreas or at the duodenum defined as the gastrinoma triangle (3). Between 60-90% of gastrinomas are malignant with metastatic spread to lymph nodes and liver (4). Gastrinoma is characterized byelevated basal serum concentration of gastrin, a hormone causing gastric acid hypersecretion. The most common presenting symptoms are abdominal pain, diarrhea and gastroesophageal reflux (2).

Somatostatin receptor scintigraphy (SRS) is a valuable method for the detection of somatostatin receptor-positive lesions (5). ^111^In Pentetreotide is a (^111^In DTPA-D-Phe) conjugate of octreotide, a somatostatin analog that binds to somatostatin receptors predominantly to receptor subtypes sst2 and sst5 (6,7). This peptide concentrates in neuroendocrine and some non-neuroendocrine tumors containing somatostatin receptors such as adrenal medullary tumors, gastroenteropancreatic tumors, carcinoid tumors, medullary thyroid carcinoma, melanoma, merkel cell tumor of the skin, paraganglioma, pituitary adenomas, and smallcell lung carcinoma. Most gastrinomas (over)-express the somatostatin receptor subtype 2 which can be targeted by ^111^In labeled Octreotide (7).

In this case report, a patient with gastrinoma metastasis to liver localized by somatostatin receptor scintigraphy is presented and the importance of SRS to show the spread of disease and to evaluate the primary lesion is emphasized.

## CASE REPORT

A 45-year-old man was admitted to the hospital suffering from epigastric pain. Multiple liver lesions were seen in abdominal ultrasonography. Percutanous liver core biopsies were made and showed metastasis of neuroendocrine carcinoma. Immunohistochemical studies of the liver tumor cells were positive for keratin, synaptophysin, kromogranin and CD 56.

**Figure 1:**
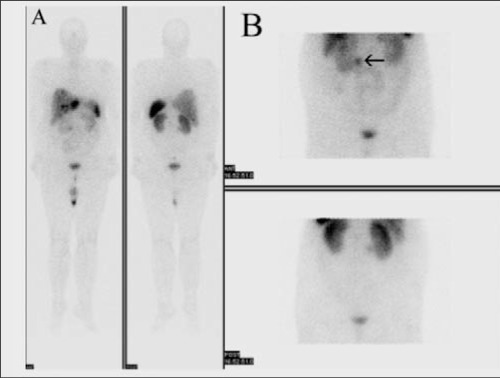
Whole-body (A) and planar (B) somatostatin-receptor scintigraphy at 5 hours show multiple intense focal uptakes of theradiotracer in the liver lesions indicating somatostatin receptor positiveliver metastasis. Planar images (B) show focal tracer uptake at theright side of the upper abdominal region (arrow)

**Figure 2:**
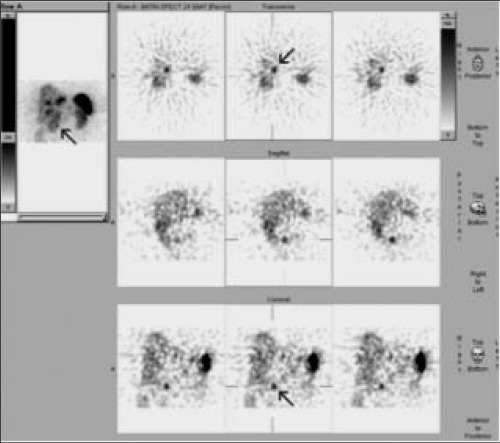
SPECT images at 24 hours show focal tracer uptake at theright side of the upper abdominal region corresponding to duodenumor the uncinate process of pancreas (arrow)

**Figure 3:**
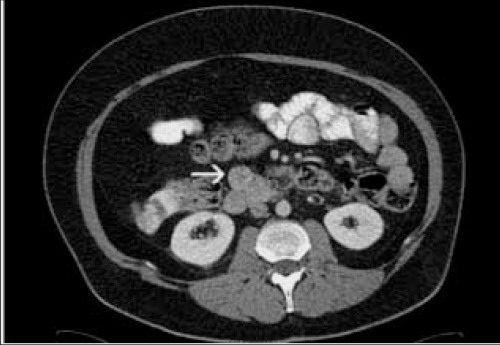
CT shows a nodular lesion approximately 2 cm in diameter(arrow)

Abdominal CT, MR imaging and SRS were performed to assess the primary tumor. SRS images were obtained 5 and 24 hours after injection of 3 mCi (^111^MBq) ^111^In- pentetreotide (OctreoScan) using dual-headed gamma camera (GE Infinia) equipped with middle energy collimators. The energy peaks were centered at 173 and 247 keV with a 20% window. Whole-body scan and planar spot views of thorax and abdomen at 5 hours and planar and SPECT images of abdomen were obtained at 24 hours. SPECT images were acquired using a circular orbit (in 128^128 matrix, 64 images, 40 s/step). The images were reconstructed using a standard filtered back projection algorithm with Butterworth filter on the Siemens e.soft workstation. Whole-body images showed multiple intense focal uptakes of the radiotracer in the liver indicating somatostatin receptor positive liver metastasis (Figure 1a). Planar and SPECT images also showed focal tracer uptake at the right side of the upper abdominal region corresponding to duodenum or the uncinate process of pancreas (Figure 1b, 2). CT and MR showed a nodular lesion with approximately 2 cm in diameter (Figure 3). MR imaging also demonstrated an ulcer focus or microperforation at proximal duodenum and jejunum.

Laboratory results were negative for the tumor markers such as CA 125, CA 19-9, CEA, but demonstrated a high blood gastrin level (1300 pc/ml; normal <125 pc/ml). The patient underwent upper gastrointestinal truck endoscopy which showed increased gastric folds and multiple ulcerations. Gastric pH was 4. In addition, laboratory studies depicted elevated serum parathormone (349 pg/ml; normal=10-70 pg/ml). Serum calcium level was 10.67 mg/dl (normal levels=8.40-10.20 mg/dl). Parathyroid scan with Tc-99m MIBI showed a slight tracer uptake at right inferior pole of the thyroid gland and parathyroid ultrasonography showed a suspicious 7x4 mm hypoechoic lesion at the same region. MR imaging of the hypophysis showed a 5x4 mm lesion at the left side of the hypophysis gland. The serum levels of TSH, prolactin, FSH, LH, cortisol, ACTH and GH were normal, indicating a nonsecretory adenoma.

Based on these results, it is concluded that the patient had MEN 1 including gastrinoma, primary hyperparathyroidism and non-secretory pituitary adenoma. As MEN 1 is an autosomal-dominant disorder, the patient and his family are recommended to have genetic evaluation. The patient refused surgery and local therapy to liver lesions. Proton pump inhibitors (lansaprazol 30 mg/twice a day) and long acting somatostatin treatment (30 mg/monthly i.m.) were started.

## DISCUSSION

Multiple endocrine neoplasia type 1 (MEN1) syndrome includes varying combinations of more than 20 endocrine and non-endocrine tumors. Endocrine tumors become symptomatic by overproduction of hormones by the tumor or by growth of the tumor itself. Clinical diagnostic criteria for MEN1 syndrome include the presence of two endocrine tumors that are parathyroid, pituitary, or gastro- entero-pancreatic tract tumors. Biochemical testing and imaging detect pituitary, parathyroid and pancreatic tumors (2). In our case, high serum concentrations of parathormone and gastrin levels were found. MR showed pituitary adenoma. Ultrasonography and parathyroid scintigraphy showed suspicious parathyroid adenoma. CT, MR and SRS were used to evaluate primary lesion and liver metastasis.

Somatostatin receptor scintigraphy can be used to localize neuroendocrine tumors with a good sensitivity and has been proven to significantly contribute to patient management. The sensitivity of SRS depends on the size and localization of the tumors. The advantages of SRS are its ability to localize tumor throughout the body at one time and demonstrate both the primary and metastatic tumors. Small lesions and tumors located in the duodenum show a lower sensitivity. Endoscopic ultrasonography (EUS) is the most sensitive imaging procedure for the detection of small (<10 mm) pancreaticoduodenal endocrine tumors in MEN-1, whereas SRS is the procedure of choice for the identification of metastases of MEN-1 pancreaticoduodenal endocrine tumors for staging (8). Positron emission tomography (PET) with 68Ga labeled octreotide analogues is increasingly used with a good accuracy (9). PET with 18F-deoxy-glucose (FDG) can be used for the detection and staging of a variety of tumors, but its sensitivity is low in well differentiated tumors such as neuroendocrine tumors (10).

More than 90% of gastrinomas are malignant. The spectrum of clinical disease progression includes localized tumors, regional lymph node metastases, and widespread metastatic disease (4). Liver lesions can be detected with high sensitivity by SRS (11). Gastrinomas have high densities of somatostatin receptors, which can be used to image these tumors using radiolabeled somatostatin analogues. 68Ga DOTATOC/TATE-PET scan is the most sensitive type of somatostatin receptor scintigraphy that detects somatostatin receptor-positive tumors and metastasis (9).

Of all imaging modalities, EUS followed by SRS is the most sensitive modality for the assessment of pancreatic tumors in asymptomatic patients suffering from a MEN-I syndrome. Scintigraphy has the highest sensitivity for tumors of symptomatic patients and for the assessment of metastases. CT and MR are only second line diagnostic modalities (12).

MEN 1 syndrome-associated malignancy currently account for approximately 30% of deaths in MEN 1 syndrome (2). Accurate preoperative localization of duodenum gastrinomas helps optimize surgical management. Surgery is the main treatment strategy. Depending on the location of the tumor in the duodenum, surgical options vary from enucleation to localized resection to pancreaticoduodenectomy (13,14) Proton pump inhibitors (PPI) are used for gastric acid hypersecretion. In advanced disease, chemotherapy, somatostatin analogues, hepatic embolization, radiotherapy and liver transplantation are the treatment options. Our patient recieved proton pump inhibitors and long acting somatostatin treatment.

In summary, we presented a patient admitted with multiple neuroendocrine liver metastases. The primary tumor was located by SRS in a region corresponding to duodenum or the uncinate process of pancreas. Other imaging techniques, CT and MR, contributed to SRS showing a nodular lesion in the same location. Serum gastrin level confirmed the diagnosis of gastrinoma. We would like to present this case to revive the role of somatostatin receptor scintigraphy in the assessment of primary tumor in a patient diagnosed with neuroendocrine liver metastases.
